# Timing of birth for women with a twin pregnancy at term: a randomised controlled trial

**DOI:** 10.1186/1471-2393-10-68

**Published:** 2010-10-25

**Authors:** Jodie M Dodd, Caroline A Crowther, Ross R Haslam, Jeffrey S Robinson

**Affiliations:** 1 Australian Research Centre for the Health of Women and Babies (ARCH), Discipline of Obstetrics and Gynaecology, The University of Adelaide, Adelaide, Australia; 2 Department of Perinatal Medicine, The Women's and Children's Hospital, Adelaide, Australia

## Abstract

**Background:**

There is a well recognized risk of complications for both women and infants of a twin pregnancy, increasing beyond 37 weeks gestation. Preterm birth prior to 37 weeks gestation is a recognized complication of a twin pregnancy, however, up to 50% of twins will be born after this time.

The aims of this randomised trial are to assess whether elective birth at 37 weeks gestation compared with standard care in women with a twin pregnancy affects the risk of perinatal death, and serious infant complications.

**Methods/Design:**

Design: Multicentred randomised trial.

Inclusion Criteria: women with a twin pregnancy at 36^6 ^weeks or more without contraindication to continuation of pregnancy.

Trial Entry & Randomisation: Following written informed consent, eligible women will be randomised from 36^+6 ^weeks gestation. The randomisation schedule uses balanced variable blocks, with stratification for centre of birth and planned mode of birth. Women will be randomised to either elective birth or standard care.

Treatment Schedules: Women allocated to the elective birth group will be planned for elective birth from 37 weeks gestation. Where the plan is for vaginal birth, this will involve induction of labour. Where the plan is for caesarean birth, this will involve elective caesarean section. For women allocated to standard care, birth will be planned for 38 weeks gestation or later. Where the plan is for vaginal birth, this will involve either awaiting the spontaneous onset of labour, or induction of labour if required. Where the plan is for caesarean birth, this will involve elective caesarean section (after 38 and as close to 39 weeks as possible).

Primary Study Outcome: A composite of perinatal mortality or serious neonatal morbidity.

Sample Size: 460 women with a twin pregnancy to show a reduction in the composite outcome from 16.3% to 6.7% with adjustment for the clustering of twin infants within mothers (p = 0.05, 80% power).

**Discussion:**

This is a protocol for a randomised trial, the findings of which will contribute information about the optimal time of birth for women with an uncomplicated multiple pregnancy at and beyond 37 weeks gestation.

**Clinical Trial Registration:**

Current Controlled Trials ISRCTN15761056

## Background

### Twin pregnancies and perinatal mortality and morbidity

It is well established that the risks associated with a twin pregnancy are greater for both mother and infants when compared with singleton pregnancies. The increased risk of stillbirth in twin pregnancies has been long recognised, with a literature report from the Dublin Lying-in Hospital dating to 1784, noting that "one-half more twins die and near one-third more are stillborn, than of single children"[1]. A number of more recent studies have evaluated the association between advancing gestational age and risk of morbidity and mortality in multiple pregnancies [2-7].

We examined retrospective data from the South Australian Pregnancy Outcome Unit, from 1991-2000, and found that the stillbirth rate for twin pregnancies was significantly higher than for singletons at each week of gestational age (p < 0.0001) [4]. There was an increase in stillbirth rate for singleton pregnancies, from 0.79 per 1000 births at 40 weeks gestation, to 3.1 per 1,000 births at 42 weeks gestation. A similar trend was noted with twin pregnancies, but was seen at an earlier gestational age, rising from 36 weeks [4]. Similarly, infants of twin pregnancies had significantly higher neonatal mortality rates than singleton infants at all gestational ages of birth (p < 0.0001), with an increase evident from 35 weeks gestation, and reaching a maximum at 40 weeks gestation of 6.49 per 1,000 live births, almost 15 times greater than that observed in singletons at the same gestational age [4].

Reported neonatal outcomes consistent with hypoxia have included Apgar score of less than 7 at 5 minutes, birth weight less than the third centile for gestational age and sex [8], cord pH at birth of less than 7.00 [9], admission to the neonatal intensive care unit (NICU), need for ventilatory assistance, seizures within the first 24 hours of life, need for tube feeding and a diagnosis of hypoxic ischaemic encephalopathy by ultrasound. These adverse outcomes are those considered by experts as important measures of term and post-term morbidity [10]. When evaluating these measures as a composite morbidity index, infants of twin pregnancies were found to have a greater frequency of adverse outcomes at each week of gestational age than infants of singleton pregnancies, being lowest at 37 weeks gestation (20.7 per 1,000) before increasing [4].

Luke and colleagues [6] retrospectively reviewed 163 twin pregnancies, and developed several models of the "ideal twin pregnancy". Using multivariate logistic regression, the best model of intrauterine growth and lowest perinatal morbidity was at an earlier gestation for twins than for singletons. Using length of stay and growth restriction criteria, 70 percent of "ideal" twin pregnancies delivered between 35 and 38 weeks gestation [6].

Cincotta and colleagues [3] retrospectively reviewed data from Queensland (Australia), over a 10 year period from 6,328 women with a twin pregnancy, to establish the gestational age-specific stillbirth risk for both twins and singleton gestations. On the basis of this information, the authors concluded that the gestation-specific rise in stillbirth rate seen in singletons at 40 weeks and beyond occurs in twins from 36 weeks gestation and onwards [3].

Minakami and Sato [7] have suggested that the estimated date of confinement in twin pregnancies is between 37 and 38 weeks gestation. This is based on retrospective information obtained from almost 89,000 infants born to women with a twin pregnancy in Japan between 1989 and 1993. This study found a mean gestation at birth for twins of 37 weeks, with the risk of stillbirth and early neonatal death increasing after 38 weeks gestation. The lowest risk of perinatal death in twin pregnancies at 38 weeks gestation corresponded to that observed in singleton pregnancies at 43 weeks gestation [7].

Cheung and colleagues [2] obtained similar data from the Swedish Medical Birth Registry for twin gestations delivered between 1982 and 1995. The models used identified a higher mortality rate among twins born after 37 weeks when compared with singleton infants at similar gestational age [2].

Hartley and colleagues [5] retrospectively analysed the birth and death certificates, and hospital discharge data for 8,150 twin pairs born in Washington State between 1987 and 1997. The lowest perinatal mortality rate for twin gestations was found with birth at 37 weeks gestation [5].

These studies indicate an increase in risk of stillbirth in twin pregnancy with advancing gestational age, the lowest risk of perinatal mortality and morbidity being observed with birth between 36 and 38 weeks gestation [2,3,5-7].

### Benefits of induction of labour

Elective induction of labour has been proposed in a number of clinical situations, with the aim of reducing adverse outcomes for both women and their infants. Induction of labour after 41 weeks gestation in women with a singleton pregnancy has been shown to be associated with a reduction in perinatal mortality [11]. Retrospective studies indicate that the risk of stillbirth and early neonatal death in a twin gestation correlates with that seen beyond 41 weeks in a singleton gestation [2,3,5-7]. The question to then be considered relates to prospectively defining the "post-term" twin pregnancy, and to assess the role of induction of labour for women with a post-term twin pregnancy in reducing perinatal mortality. The potential advantages of elective timing of birth in women with a twin pregnancy at 37 weeks' gestation of a reduction in perinatal mortality and morbidity have to be balanced against any associated increase in the risk of caesarean section and potential risks for the infants associated with early birth, including respiratory distress syndrome and need for admission to the neonatal unit.

### Evidence for the optimal timing of birth for women with a twin pregnancy at term: a systematic review of the literature

In a Cochrane Systematic Review, we reviewed the literature to assess whether a policy of elective delivery from 37 weeks' gestation compared with an expectant approach for women with an otherwise uncomplicated twin pregnancy was associated with improved infant outcome [12]. A single randomised controlled trial was included addressing the role of induction of labour versus expectant management for women with a twin pregnancy from Japan [13].

This trial, recruited 36 women with a twin pregnancy, who gave birth after 37 weeks gestation, where the first twin was in a cephalic presentation [13]. Nineteen women were randomised to the expectant management group that consisted of daily evaluation by non-stress cardiotocograph (CTG), and twice weekly ultrasound examination. The onset of spontaneous labour was awaited provided no complications developed and fetal well-being was confirmed. Seventeen women were randomised to induction of labour, involving intravaginal prostaglandin E_2 _followed by amniotomy and oxytocin infusion when feasible. There were no losses to follow-up. The authors did not describe the method of randomisation and allocation concealment.

There were no statistically significant differences identified in baseline characteristics between the two study groups [13]. The average gestational age at birth in the induction group was 37.5 +/- 0.4 weeks, significantly earlier than in the expectant management group (39.0 +/- 1.1 weeks; p < 0.05). Seven women in the expectant management group had prelabour rupture of the membranes (PROM), and five neonates in this group had meconium stained liquor. In comparison, no women in the induction of labour group developed PROM (p < 0.05), and no infants were noted to have meconium stained liquor (p < 0.05). There were no statistically significant differences identified between the two treatment groups with regards to mode of birth, infant birth weight of less than 2500 grams, or Apgar score of less than 7 at 5 minutes of age [13].

While the results of this trial [13] indicate no statistically significant differences between elective induction of labour at 37 weeks' gestation and continued expectant management for the outcomes indicated, the small sample size means that it was underpowered to detect differences in the clinical outcomes of interest, and further information is required. On the basis of this single randomised controlled trial, there are insufficient data available to support a practice of elective delivery from 37 weeks gestation for women with an otherwise uncomplicated twin pregnancy at term.

### To summarise

Up to 50% of women with a twin pregnancy (approximately 1800 women in Australia per annum) will reach 37 weeks gestation and beyond. The risk of perinatal mortality (stillbirth and neonatal death) and neonatal morbidity associated with twin pregnancies has been demonstrated to increase with advancing gestational age. While elective birth at 37 weeks gestation may be a safe and effective way to reduce the perinatal mortality and morbidity in twins, the current evidence on the role of elective birth at 37 weeks gestation for women with a twin pregnancy is limited.

#### Aims of the trial

The aims of this randomised controlled trial are to assess whether a policy of elective birth at 37 weeks gestation compared with a policy of standard management in women with a twin pregnancy affects the risk of perinatal death (stillbirth or neonatal death); and the risk of serious complications for the infant.

#### Trial hypotheses

**The primary hypothesis **of this randomised trial is that for women with a twin pregnancy elective timing of birth at 37 weeks gestation is associated with

• **a reduction in serious adverse outcome for the infant**, defined as one or more of stillbirth, neonatal death or significant infant morbidity.

**The secondary hypotheses **of the trial are that for women and infants of a twin pregnancy elective timing of birth at 37 weeks gestation compared with standard management is associated with

• a reduction in antenatal medical and obstetric complications

• a reduction in labour and birth complications

• a reduction in other infant complications

## Methods and design

### Study Design

Multicentred randomised controlled trial

### Inclusion Criteria

All women with a twin pregnancy at gestational age of 36^6 ^weeks or more without a contraindication to continuation of pregnancy, presenting to the antenatal clinic or ward of participating collaborating centres will be eligible for trial participation.

### Exclusion Criteria

Women with any of the following will be excluded from the trial: intrauterine fetal death of one or both fetuses at the time of trial entry; active labour; fetal distress or non-reassuring fetal heart rate trace; maternal or fetal compromise precluding continued antenatal surveillance.

### Trial Entry

At the first antenatal clinic or upon diagnosis, all women with a twin pregnancy will be given the trial information sheet, counselled by a member of the research team and encouraged to discuss the study with her family. A member of the research team will then obtain provisional, written, informed consent. To maximise the likelihood that women will receive the care allocated at randomisation, eligible women will be randomised from 36^6 ^weeks gestation. Eligibility and consent will be reconfirmed just prior to randomisation.

After the woman is eligible to be randomised, entry details will be recorded on the trial entry form, and the central telephone randomisation service at the Australian Research Centre for Health of Women and Babies, Discipline of Obstetrics and Gynaecology, University of Adelaide will be contacted. During a short telephone call, information to check eligibility will be sought that, describes the woman, enables stratification at randomisation so that similar women are allocated to the treatment arms, assists in follow-up and assists in the analysis of results. If an eligible woman does not agree to randomisation, minimal details will be recorded to complete the requirements for reporting of clinical trials (CONSORT).

Once all entry details are given at telephone randomisation and eligibility confirmed, a study number will be allocated to the woman by the central randomisation office, and the group to which the woman is allocated (either elective birth or standard care) will be stated. An investigator not involved with the treatment allocation of the women, using balanced variable blocks, will prepare the randomisation schedule. Stratification will be by collaborating centre and planned mode of birth (planned caesarean section or planned vaginal birth).

### Elective Birth Group

Women allocated to the elective birth group will be planned for elective birth from 37 weeks gestation. Where there is a plan for vaginal birth, this will involve induction of labour. Where there is a plan for caesarean birth, this will involve an elective caesarean section. The planned mode of birth will be assessed and determined by the woman and the obstetrician caring for her.

### Standard Care Group

For women allocated to the standard care group, birth will be planned for 38 weeks gestation or later, according to the hospital where they expect to give birth. Where there is a plan for vaginal birth, this will involve either awaiting the spontaneous onset of labour, or induction of labour if required. Where there is a plan for caesarean birth, this will involve an elective caesarean section (where possible booked after 38 weeks and as close to 39 weeks gestation as possible). The planned mode of birth will be assessed and determined by the woman and the obstetrician caring for her.

If earlier birth (before 38 weeks gestation) is considered appropriate due to the development of complications, this will be carried out either by induction of labour or by caesarean section, as determined by the woman and the obstetrician caring for her. However, the woman will remain in the standard care group for the purposes of analysis.

### Care of Women in Both Groups

Ongoing assessment of fetal well-being will be provided according to the local hospital guidelines where the woman is planned to give birth. The process of induction of labour will be carried out according to the usual practices of the attending obstetrician and the hospital involved. All women are recommended to have continuous fetal heart rate monitoring by cardiotocograph (CTG) when in active labour. The presence of a non-reassuring fetal heart rate tracing will be managed by performing fetal scalp pH sampling to assist decision making where possible, or emergency caesarean section as appropriate. The spectrum of analgesia and anaesthesia should be available according to the woman's choice.

In order to meet these requirements for care of women participating in the trial, each collaborating centre should be able to perform continuous electronic fetal heart rate monitoring during labour, be able to perform fetal scalp pH sampling in the setting of a non-reassuring fetal heart rate trace, have on-site skilled obstetric, anaesthetic and paediatric staff, be able to perform an emergency caesarean section, have an available obstetric consultant for emergency back-up, and be able to cross match blood. Care for the woman will be managed by the obstetric team with care of the infant by the attending neonatologist.

### Follow up of Women in Both Groups

After birth, information will be obtained relating to birth and infant outcomes from the woman and infants' case notes by the research assistant. The delivery form will be completed after the woman has given birth. Similarly, the postnatal and neonatal forms will be completed for each live born infant after discharge of both mother and infants from hospital. After birth, placental pathology will be performed to confirm the chorionicity of the pregnancy.

### Primary Study Endpoint

A composite mortality and morbidity index has been chosen as the primary outcome for the trial. For a policy of elective birth at 37 weeks gestation to be justified in clinical practice, there must be an important benefit of reduced perinatal mortality or serious adverse outcome for the infants defined as one or more of the following:

• **Perinatal mortality**defined as *any fetal death after trial entry, or death of a liveborn infant within 28 days of age (excluding lethal congenital anomalies); *or

• **Serious neonatal morbidity**defined as one or more of the following, excluding lethal congenital anomalies, and reflecting either **asphyxia **(*birth trauma *(subdural or intracerebral haemorrhage, spinal cord injury, basal skull fracture, other fracture, peripheral nerve injury present at discharge from hospital); *birth weight ≤ 3^rd ^centile for gestational age at birth and infant sex *[8]; *Apgar score ≤ 4 at 5 minutes of age; cord pH ≤ 7.00 *[9]; *seizures at ≤ 24 hours age or requiring two or more drugs to control; neonatal encephalopathy grade 3 or 4 *[14]) or **immaturity **(*use of ventilation ≥ 24 hours; admission to neonatal intensive care unit (NICU) ≥ 4 days; severe respiratory distress syndrome *(MAP ≥ 10 and or FiO_2 _≥ 0.8 with need for ventilation)*; neonatal encephalopathy grade 3 or 4 *[14]*chronic lung disease defined as continued oxygen requirement at 28 days of life; proven necrotising enterocolitis; proven systemic infection within 48 hours of birth treated with antibiotics).*

These definitions of adverse outcome are those used by the Australian and New Zealand Neonatal Network,[15] and those considered by experts as important measures of term and post-term neonatal morbidity [10].

### Secondary Study Endpoints

*1. ***Antenatal medical and obstetric complications**including: pre-eclampsia or eclampsia *(systolic blood pressure ≥ 140 mmHg and diastolic ≥ 90 mmHg on two occasions four hours apart or more, and one of proteinuria (≥ 300 mg/24 hours or spot urine creatinine ratio ≥ 30 mg/mmol), renal insufficiency (serum plasma creatinine ≥ 0.09 mmol/L or oliguria), liver disease (elevated serum transaminases &/or right upper quadrant pain), neurological disturbances (convulsions, hyperreflexia with clonus, severe headache with hyperreflexia, persistent visual disturbances), or haematological disturbances (thrombocytopaenia, disseminated intravascular coagulopathy, haemolysis),)*[16] antepartum haemorrhage requiring hospitalisation; and abnormal umbilical artery Doppler study *(absent or reversed end diastolic flow as detected by ultrasound examination).*

2. **Labour and birth complications**including: induction of labour for medical or obstetric complications; meconium stained liquor; cardiotocogram (CTG) abnormality during labour (*fetal tachycardia (fetal heart rate greater than 160 bpm); fetal bradycardia (fetal heart rate less than 110 bpm); reduced variability (less than 5 bpm); decelerations of the fetal heart rate (early, late or variable))*; instrumental vaginal birth; emergency caesarean birth (*all and for fetal distress).*

3. **Adverse outcomes for the infant**defined as: Apgar score <7 at 5 minutes; birthweight less than 10^th ^centile for gestational age and infant sex; admission to neonatal intensive care unit (NICU) and length of stay; incidence and severity of any neonatal respiratory disease; use of and length of any mechanical ventilation; use of and length of any tube feeding.

4. **Composite of serious neonatal outcomes reflecting asphyxia**defined as one or more of: *death due to asphyxial causes*; *birth trauma *(subdural or intra-cerebral haemorrhage, spinal cord injury, basal skull fracture, other fracture, peripheral nerve injury present at discharge from hospital); *birth weight ≤ 3*^*rd *^*centile for gestational age at birth and infant sex *[8]; *Apgar score ≤ 4 at 5 minutes of age; cord pH ≤ 7.00 *[9]; *seizures at ≤ 24 hours age or requiring two or more drugs to control; neonatal encephalopathy grade 3 or 4 *[14]).

5. **Composite of serious neonatal outcomes reflecting immaturity**defined as one or more of: *death due to immaturity causes; use of ventilation ≥ 24 hours; admission to neonatal intensive care unit (NICU) ≥ 4 days; severe respiratory distress syndrome *(MAP ≥ 10 and or FiO_2 _≥ 0.8 with need for ventilation)*; neonatal encephalopathy grade 3 or 4 *[14]*; chronic lung disease defined as continued oxygen requirement at 28 days of life; proven necrotising enterocolitis; proven systemic infection within 48 hours of birth treated with antibiotics.*

### Sample Size

The incidence of serious adverse infant outcome is the principal endpoint of the trial. Using a composite neonatal morbidity and mortality index based on perinatal mortality, admission to NICU for greater than or equal to four days, and birthweight less than the third centile for gestational age at birth and infant sex, the occurrence of serious adverse neonatal outcome for infants of a twin pregnancy born at 37 weeks gestation or more is estimated at 16.3% [4]. To reduce this to the rate of 6.7% seen in singleton infants at the same gestational age [4], and adjusting for the clustering of twin infants within mothers, a sample size of 460 women with a twin pregnancy at ≥ 37 weeks gestation will be required, using a p-value of 0.05 (two tailed) and power 80%.

## Analysis and Reporting of Results

We will conduct an interim analysis when we have recruited 50% of the total sample size (230 women).

The initial analysis will examine the baseline characteristics of all randomised women, as an indication of comparable treatment groups, and include maternal age, race, height, weight, smoking history, past obstetric history (including previous perinatal loss), and chorionicity of the pregnancy. Any important differences in these prognostic variables will be controlled for in subsequent analyses. Outcome comparisons for women and infants will be analysed for the primary and secondary outcomes on an "intention to treat" basis, according to treatment allocation at randomisation. Continuous outcomes will be analysed using linear mixed model regression. Binary outcomes will be analysed using a generalised linear mixed model with a log link function and binomial distribution. A random effect to account for clustering of babies from multiple births will be included. If models do not converge, then alternative methods of accounting for multiple births using robust variance estimation will be explored. Results will be presented as relative risks with 95% confidence intervals. The level of significance will be 0.05 and all p values will be two-sided.

Pre-specified subgroup analyses will be by chorionicity of the pregnancy (monochorionic or dichorionic); collaborating centre; and actual mode of birth.

Approval to conduct this study has been obtained from the following research and ethics committees: Women's and Children's Hospital (Adelaide, South Australia); Lyell McEwin Health Service (Adelaide, South Australia); The Royal Women's Hospital (Melbourne, Victoria); Sydney West Area Health Service (New South Wales); Northern Sydney Central Coast (New South Wales); Mater Health Services (Brisbane, Queensland); Redcliffe-Caboolture Health Service (Queensland); Logan Hospital (Queensland); Townsville Health Service (Queensland); Mackay Base Hospital (Queensland); and Auckland City Hospital (New Zealand).

## Discussion

This is a protocol for a randomised trial assessing a policy of elective birth at 37 weeks gestation versus a policy of continued expectant care for women with an uncomplicated twin pregnancy at term. The findings of this trial will contribute to the currently available literature regarding the optimal time of birth for women with an uncomplicated multiple pregnancy at and beyond 37 weeks gestation.

## Competing interests

The authors declare that they have no competing interests.

## Authors' contributions

JMD, CAC, RRH, JSR all contributed to the development of the trial protocol. JMD drafted this manuscript; all authors reviewed critically for content and gave approval to the final to be published version of this manuscript.

## Pre-publication history

The pre-publication history for this paper can be accessed here:

http://www.biomedcentral.com/1471-2393/10/68/prepub

## References

[B1] Clarke J Observations of the excess of the mortality of males above that of females 1786

[B2] Cheung YB Yip P Karlberg J Mortality of twins and singletons by gestational age: a varying-coefficient approach Am J Epidemiol 2000 152 12 1107 1116 10.1093/aje/152.12.1107 11130615

[B3] Cincotta R Flenady V Hockey R King J Mortality of twins and singletons by gestational age: a varying coefficient approach Perinatal Society of Australia and New Zealand, 5th Annual Congress: 2001; Canberra, Australia 2001 22

[B4] Dodd J Robinson J Crowther C Chan A Stillbirth and neonatal outcomes in South Australia, 1991-2000 Am J Obstet Gynecol 2003 189 1731 1736 10.1016/S0002-9378(03)00854-8 14710106

[B5] Hartley R Emanuel I Hitti J Perinatal mortality and neonatal morbidity rates among twin pairs at different gestational ages: optimal delivery timing at 37-38 weeks' gestation Am J Obstet Gynecol 2001 184 451 458 10.1067/mob.2001.109399 11228502

[B6] Luke B Minogue J Witter F Keith L Johnson T The ideal twin pregnancy: patterns of weight gain, discordance, and length of gestation Am J Obstet Gynecol 1993 169 3 588 597 8372868 10.1016/0002-9378(93)90628-v

[B7] Minakami H Sato I Re-estimating the date of delivery in multifetal pregnancies JAMA 1996 275 18 1432 1434 10.1001/jama.275.18.1432 8618370

[B8] Roberts CL Lancaster PAL Australian national birthweight percentiles by gestational age Medical Journal of Australia 1999 170 114 118 10065122 10.5694/j.1326-5377.1999.tb127678.x

[B9] ACOG ACOG Committee Opinion No. 348, November 2006: Umbilical cord blood gas and acid-base analysis Obstet Gynecol 2006 108 5 1319 1322 17077266 10.1097/00006250-200611000-00058

[B10] Hannah ME Hannah WJ Hellmann J Induction of labor as compared with serial antenatal monitoring in post-term pregnancy. A randomised controlled trial NEJM 1992 326 1587 1592 10.1056/NEJM199206113262402 1584259

[B11] Gulmezoglu AM Crowther CA Middleton P Induction of labour for improving birth outcomes for women at or beyond term Cochrane Database of Systematic Reviews 2006 4 CD004945 10.1002/14651858.CD004945.pub2 17054226

[B12] Dodd JM Crowther CA Elective delivery from 37 weeks gestation in women with a twin pregnancy (Cochrane Review) The Cochrane Database of Systematic Reviews 2003 1 10.1002/14651858.CD003582 12535480

[B13] Suzuki S Otsubo Y Sawa R Yoneyama Y Araki T Clinical trial of induction of labor versus expectant management in twin pregnancy Gynecol Obstet Invest 2000 49 1 24 27 10.1159/000010207 10629368

[B14] Sarnat HB Sarnat MS Neonatal encephalopathy following fetal distress Archives of Neurology 1976 33 696 705 987769 10.1001/archneur.1976.00500100030012

[B15] Donoghue D Cust A Australian and New Zealand Neonatal Network 1998 2000 Australian Institute of Health and Welfare NPSU. Sydney

[B16] Brown MA Hague WM Higgins J The detection, investigation and management of hypertension in pregnancy Aust NZ J Obstet Gynaecol 2000 4 2 139 155 10.1111/j.1479-828X.2000.tb01137.x 10925900

